# Preferences of physicians for public and private sector work

**DOI:** 10.1186/s12960-020-00498-4

**Published:** 2020-08-10

**Authors:** Anthony Scott, Jon Helgeim Holte, Julia Witt

**Affiliations:** 1 Melbourne Institute: Applied Economic and Social Research, The University of Melbourne, Level 5 FBE Building, 111 Barry Street, Melbourne, VIC 3010 Australia; 2 FAFO Institute for Labour and Social Research, Borggata 2B, 0608 Oslo, Norway; 3 Department of Economics, University of Manitoba, Winnipeg, MB R3T 5V5 Canada

**Keywords:** Public sector, Dual practice, Physicians, Discrete choice experiments, Risk aversion

## Abstract

**Background:**

The public-private mix of healthcare remains controversial. This paper examines physicians’ preferences for public sector work in the context of dual practice, whilst accounting for other differences in the characteristics of jobs.

**Methods:**

A discrete choice experiment is conducted with data from 3422 non-GP specialists from the Medicine in Australia: Balancing Employment and Life (MABEL) panel survey of physicians.

**Results:**

Physicians prefer to work in the public sector, though the value of working in the public sector is very small at 0.14% of their annual earnings to work an additional hour per week. These preferences are heterogeneous. Contrary to other studies that show risk averse individuals prefer public sector work, for physicians, we find that those averse to taking career or clinical risks prefer to work in the private sector. Those with relatively low earnings prefer public sector work and those with high earnings prefer private sector work, though these effects are small.

**Conclusions:**

Other job characteristics are more important than the sector of work, suggesting that these should be the focus of policy to influence specialist’s allocation of time between sectors.

## Background

In many healthcare systems, physicians are able to combine work in the public and private sector. Dual practice can be controversial in the context of universal health care that usually aims to provide care that does not depend on ability to pay but the need for health care [1]. Physicians choose to allocate their time across both sectors based on the institutional setting that governs regulations and remuneration, specialty, and conditional on this, physician’s preferences for the characteristics of each work setting. In a mixed public-private system, and where the number of physicians is relatively fixed in the short to medium term because of barriers to entry, physician’s preferences for the amount of time spent in each sector can influence access to health care, including public hospital waiting times, as well as expenditures and patient’s health outcomes.

The aim of this paper is to examine physician’s preferences for working in the public or private sector within a given institutional setting that allows dual practice and a rich mix of public and private health care provision and financing. The literature on physician dual practice is largely theoretical and concerned with the nature of the regulation of public and private sector work [1–4]. The drivers of physician’s choices, such as the role of job characteristics, have not yet been examined in detail. Johannessen and Hagen [5] examine associations with physician characteristics such as debt and family size, but did not examine job characteristics. Cheng et al. [6] and Saether [7] focus on the role of earnings on sector choice. We build on these studies by using a discrete choice experiment to capture a much richer set of job characteristics [8, 9]. We also examine risk attitudes which have been suggested as a reason why workers prefer the public sector to the private sector or to become self-employed [10–14] but have not been examined in the context of physician dual practice.

## Methods

### Setting

There were 1325 hospitals in Australia in 2016/2017, including 630 private hospitals. Thirty-five percent of beds and 60% of separations were in public hospitals, whilst 59% of all elective surgery is conducted in private hospitals [15]. Medicare is Australia’s national tax-financed universal health insurance scheme that subsidises out of hospital medical services (GPs and non-GP specialists), pharmaceuticals, and around 40% of funding for public hospitals. States own and run public hospitals and fund the remainder from their own tax revenues. The government indirectly subsidises activity in private hospitals through subsidies for private health insurance premiums and through Medicare rebates for patients treated in private hospitals. Around 45% of the population has private health insurance.

Non-GP specialists in Australia can be employed on a salary in public hospitals providing inpatient or outpatient services and, for those who have ‘rights to private practice’, can at the same time be self-employed and organised into small businesses. In these private settings, they work in private hospitals providing inpatient services, and/or in their own offices providing outpatient services, and they can treat private patients in public hospitals. Private non-GP specialists operate on a fee-for-service basis and can charge patients what the market will bear, and receive no remuneration from private hospitals. There are no price controls. Private patients can claim a fixed subsidy from Medicare, which is a fixed amount as determined in the Medicare Benefits Schedule. This fixed amount is usually lower than the fee charged, and so patients face an out of pocket cost. For patients in private hospitals, this out of pocket cost is insurable by some private health insurers (‘no gap’ cover) though patient eligibility for this cover is at the discretion of the specialist. Specialists who are primarily based in the private sector can choose to work in public hospitals as a contractor (Visiting Medical Officer) where they can be paid a fixed payment per session (typically a 4-h period) or by fee-for-service. The salaries of public hospital non-GP specialists are determined by State-level employer bargaining agreements, so salary scales are fixed, though public hospitals have discretion to pay above the award rates.

A previous study, using the same dataset as this paper, found that 48% of medical specialists combined public and private sector work, 19% worked in the private sector only, and 33% worked in the public sector only [16]. Public sector specialists are likely to be younger, to be international medical graduates, to devote a higher percentage of time to education and research, more likely to do after hours and on-call work, and more likely to travel to provide services in other areas, compared to private sector specialists. Dual practice and private sector specialists also have higher annual earnings compared to public sector specialists.

### Data

A discrete choice experiment was conducted as part of Wave 1 (2008) of the Medicine in Australia: Balancing Employment and Life (MABEL) panel survey of doctors [17]. The MABEL survey included the DCE and was sent to the population of doctors using four surveys: hospital non-specialists, doctors enrolled in specialty training programmes, general practitioners, and non-GP specialists in clinical practice in Australia in 2008 with 10 498 (19%) doctors responding. Doctors were invited using a personalised mailed letter that included a paper copy of the survey and also a username and password if they wished to fill out the survey online. Three reminders were sent. MABEL includes rich data, including annual earnings and other financial questions, work and job characteristics, hours worked, family circumstances, and geographic location. In this paper, we include doctors who filled out the ‘Specialists’ survey, which includes questions on the hours they work in the public and private sectors.

### DCE design

**Fig. 1:**
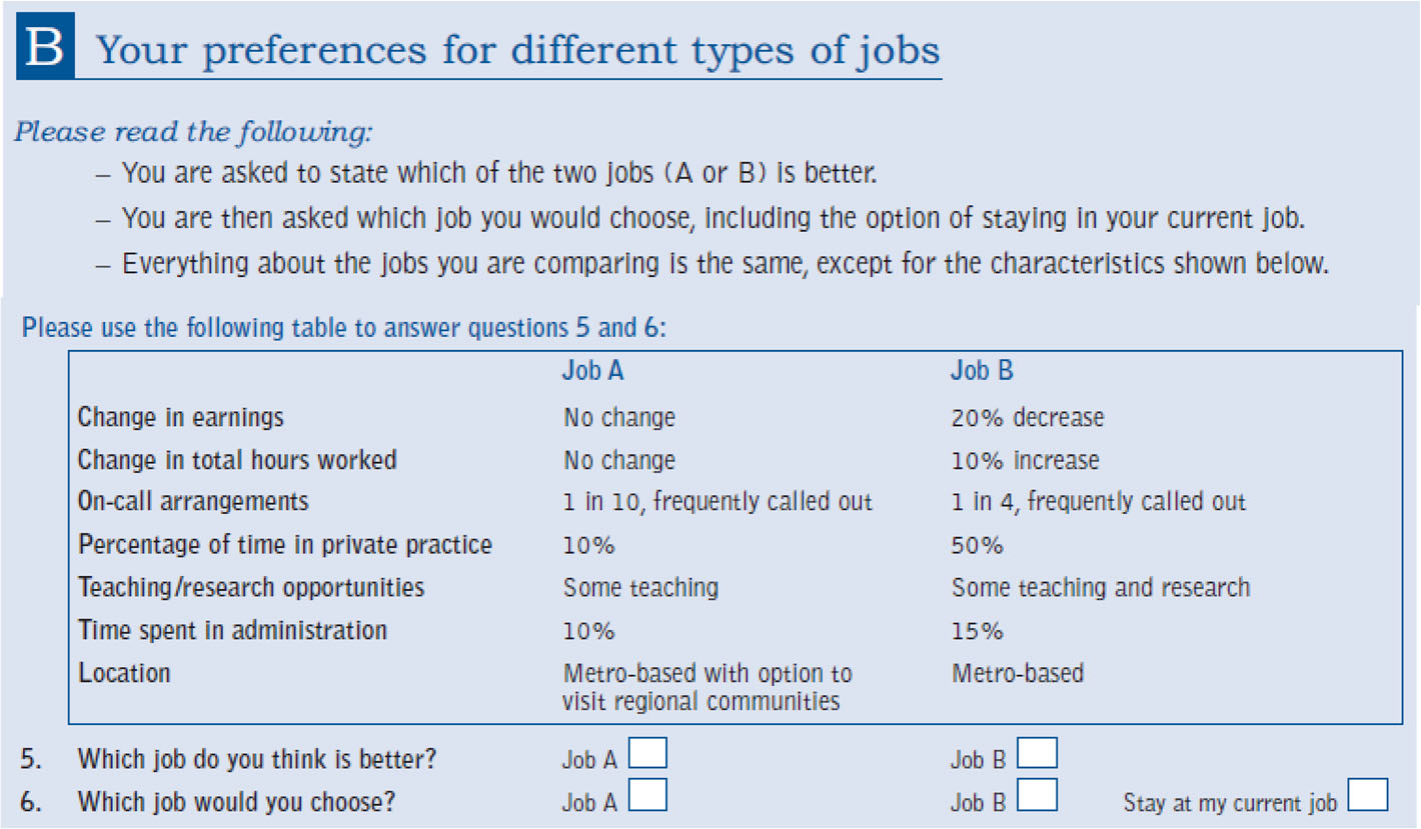
Example of DCE question

A list of attributes included in the DCE is shown in Table 1, and an example of one DCE question is shown in Fig. 1. These were informed by the existing literature and piloting and included attributes most likely to differ between sectors. The questionnaire went through four stages of piloting, including examining face and content validity of the DCEs through face to face interviews with two specialists and a meeting of 12 doctors in specialist training, and a full pilot survey.

**Table 1:** Attributes and levels

Characteristic	Levels of the characteristic
Change in earnings	20% increase
	No change
	20% decrease
Change in total hours worked	10% decrease
	No change
	10% increase
On-call arrangements	1 in 2, frequently called out
	1 in 4, frequently called out
	1 in 4, infrequently called out
	1 in 10, frequently called out
Percentage of time in private practice	10%
	50%
	90%
Teaching/research opportunities	No teaching or research
	Some teaching
	Some research
	Some teaching and research
Time spent in administration	5%
	10%
	15%
Location	Metro-based with option to visit regional communities
	Metro-based
	Large regional centre

Effects coding is used for the following attributes: on-call, teaching/research opportunities, and location. The teaching/research attribute, which consists of four levels, is dichotomised into *none* or *some* in the analysis presented here, because constructing the status quo accurately with all four levels was difficult. All the other attributes are treated as continuous variables, and a linear functional form in the respondents’ utility function is assumed

The main attribute of interest is the percentage of time spent in private practice. The levels for this attribute are 10%, 50%, and 90%. The latter (50% and 90%) are based on the average hours per week in the private sector of specialists working mainly in private practice [18], and we defined our lower bound (10%) so that specialists who work mainly in the public sector would find this realistic.

The levels of our income attributes are defined as percent changes from current income (20% increase, no change, 20% decrease) to avoid having income figures that might be unrealistic for some respondents, such as those working part time [9, 19]. Similarly, hours worked are defined in terms of changes from current hours (10% increase, no change, and 10% decrease), and these percentages were obtained from average total hours worked per week across different specialties [18, 20]. Another attribute is on-call arrangements, which is a key issue for all specialties [9, 19]. Teaching and research opportunities is included as an attribute, as these are associated with intellectual satisfaction and reputation [21]. Time spent doing administrative non-clinical work is another attribute. Any administrative time would mostly generate disutility, but specialists are likely to be willing to trade off doing some administrative work for other perks. Specialists are unlikely to work in rural and remote areas due to lack of infrastructure (i.e. hospitals). However, specialists can choose between large regional centres and metropolitan areas. Additionally, specialists can also choose to visit rural areas for short periods of time, and there are government specialist outreach programmes to support this financially.

SAS was used to generate an efficient fractional factorial experimental design [22]. Zero priors were used to generate the pilot survey as we did not have any other information. Results from the pilot surveys were used as priors in the experimental design for the main survey [23]. The experimental design produced a fractional factorial design of 36 choices which were randomly assigned to four version of the survey, each with nine choice sets.

### Analysis

The specification of the choice model is based on random utility theory where an indirect utility function is specified and estimated with three alternatives. The utility *U*_*nij*_ and choice outcome *Y*_*nij*_ of physician *n* for alternative *i* from choice set *j* is:

1 $$ {U}_{nij}={X}_{nij}\beta +{\varepsilon}_{nij} $$

for example, *Y*_*nij*_ = 1 *if U*_*n*1*j*_ > *U*_*n*2*j*_ & *U*_*n*1*j*_ > *U*_*n*3*j*_, if alternative 1 is preferred to alternatives 2 and 3

$$ n=1,\dots N;\kern0.5em j=1,\dots, J $$

where *X*_*nij*_ is a *k*-vector of observed attributes of alternative *i*, *β* is a vector of marginal utilities of the attributes, and *ε*_*nij*_ is i.i.d. extreme value and is estimated using the multinomial logit model. Two job alternatives (A and B) were presented to each doctor, and they were asked which job (A or B) they prefer (forced choice) and then asked which job they would choose: A, B, or their current job (status quo). The latter was included to account for status quo bias. In the analysis, the levels of each attribute in the status quo alternative were constructed from other questions asked in the survey that represented the doctors’ current job characteristics. For the attributes of earnings and hours worked, a zero-percentage change was used in the status quo alternative. For the percentage of time spent on administration and in the private sector, questions were asked on the actual distribution of working hours across settings (including public and private) and a separate question asked about the distribution of hours across activities (clinical, non-clinical, management and administration, education and research). The level of on-call for the status quo alternative was constructed from several questions asking whether the doctor did on-call, how many hours, and how frequently they were called out.

Unobserved heterogeneity in marginal utilities can be modelled using an extension of the multinomial logit, the mixed logit model:

2 $$ {U}_{nij}={X}_{nij}{\beta}_n+{\varepsilon}_{nij} $$

$$ {\beta}_n=\tilde{\beta}+{\eta}_n $$

where *η*_*n*_ is a vector of mean-zero individual-specific deviations from the mean marginal utility such that *β*_*n*_ is a vector of individual-specific marginal utilities of each attribute with a distribution *F(β*_*n*_*;θ)* specified by the researcher [24]. The vector of parameters *θ* (the means and standard deviations of the random coefficients *β*_*n*_) characterises the distribution of *β*_*n*_*.* We estimate a generalised multinomial logit model (G-MNL) [25], which is a mixed logit model that allows for correlation between the parameter distributions of coefficients using a single parameter [26]. Compared to a mixed logit model with uncorrelated coefficients, the GMNL models allows for correlations between the distributions of heterogeneity that is common across all coefficients due to both preference and scale heterogeneity, and so results in an improved model fit [26, 27]. Scale heterogeneity is where the variance of the error terms varies across individuals because of near-lexicographic preferences where marginal utilities for some attributes are very high (i.e. scaled up), or at the other extreme can be due to randomness of behaviour where the idiosyncratic error term dominates and an individual is very unsure of their choices. Fiebig et al. [25] argue that the G-MNL model is flexible enough to model data from these ‘extreme’ respondents, therefore providing a much better fit to the data. The GMNL model is an extension of the mixed logit model by multiplying the error term in (2) according to *1/σ*_*n*_ or equivalently by multiplying the vector of coefficients by *σ*_*n*_: ${\beta}_n={\sigma}_n\left(\overset{\sim }{\beta }+{\eta}_n\right)$ where ${\sigma}_n=\mathit{\exp}\left(\overline{\sigma}+\tau {v}_n\right)$, ${v}_n\sim N\left(0,1\right),\mathrm{and}\ \overline{\sigma}={\tau}^2/2$, so there is one extra parameter, *τ*, to be estimated. Apart from the coefficient for income, which is treated as fixed to aid the calculation of marginal rates of substitution, the coefficients for the remaining attributes are treated as random (using a normal distribution).

To examine the monetary value of private sector work (the compensating differential), we calculate the marginal rate of substitution between the earnings attribute and the private sector attribute. We also calculate these compensating earnings differentials for all other attributes using the same methods as in Scott et al. [28]. The measures of risk aversion are discussed in Additional file 1. The measures of risk aversion are interacted with the public-private attribute in the regression model to examine whether preferences for public sector work depend on risk aversion, and whether the magnitude of the marginal utility for the public-private attribute remains stable and statistically significant.

## Results

The response rate for specialists was 22.3% (4310/19 579) with a 98.4% contact rate. The final numbers of specialists who completed at least part of the DCE was 3422 with descriptive statistics in Table 2. The questionnaire was completed online by 27.6% of respondents. Respondents were broadly representative of the population of Australian specialists. MABEL respondents were slightly younger (51.2 in MABEL vs 53 years old in population), included more women specialists than the population (26.5% vs 20.6% female), were slightly less likely to come from major cities (major cities 83.7% vs 86.8%: inner regional areas 13% vs 10.6%: outer regional areas 2.7% vs 2.4%: remote areas 0.7% vs 0.2%), and worked an additional 36 min per week (44.4 vs 43.8 h per week).

**Table 2:** Characteristics of specialists responding to the DCE (*n* = 3422)

	Mean	sd	***N***	min	max
Proportion female	0.28	0.45	3422	0	1
Age	50.22	9.98	3408	31	89
Proportion with Australian medical degree	0.8	0.4	3422	0	1
Proportion with dependent children	0.69	0.46	3422	0	1
Proportion living with spouse	0.89	0.31	3134	0	1
Mean job satisfaction (5 = very satisfied)	3.21	0.82	3341	1	5
**Job characteristics**^**a**^**:**					
Annual earnings ($ before tax)^b^	325811	248106	2873	1000	3.33 m
Weekly working hours^b^	45.33	14.48	3422	0.8	120
1 in 2, frequently called out	0.19	0.39	3422	0	1
1 in 10, frequently called out	0.09	0.29	3422	0	1
1 in 4, frequently called out	0.25	0.43	3422	0	1
1 in 4, infrequently called out	0.47	0.5	3422	0	1
Proportion of time spent in private sector	0.44	0.39	3422	0	1
10% of time spent in private sector (< 30%)^c^	0.45	0.5	3422	0	1
50% of time spent in private sector (30–69%)^c^	0.20	0.4	3422	0	1
90% of time spent in private sector (> 70%)^c^	0.35	0.48	3422	0	1
No teaching or research	0.22	0.41	3422	0	1
Some teaching or research	0.78	0.41	3422	0	1
Proportion of time spent on administration	0.10	0.13	3422	0	1
5% of time spent on administration (< 7.5%)^d^	0.57	0.5	3422	0	1
10% time spent on administration (7.5–12.4%)^d^	0.17	0.37	3422	0	1
15% of time spent on administration (> 12.5%)^d^	0.26	0.44	3422	0	1
Metro-based	0.70	0.46	3422	0	1
Metro-based with option to visit regional communities	0.14	0.35	3422	0	1
Large regional centre^e^	0.16	0.37	3422	0	1

^a^Job characteristics are those that are used to construct the levels of the status quo alternative in the DCE based on questions from the MABEL survey

^b^For earnings and working hours, the level of the DCE attribute was set to 0% change

^c^For the proportion of time spent in the private sector, respondents actual time was allocated to each attribute level in bands, so for example those with less than 30% of their time in the private sector were assigned to the 10% level in the DCE to best represent their actual level

^d^For time spent on administration, respondent’s actual time was assigned to each attribute level in bands, so for example respondents who spent less than 7.5% of their time ion administration were allocated to the 5% category in the DCE

^e^This level includes all doctors in all non-metropolitan areas: 12.16% in inner regional areas, 2.78% in outer regional areas, and 0.67% in remote areas (based on Australian Standard Geographic Classification: ASGC)

Table 3 shows the number of times each alternative was chosen (out of 3422 × 9 choice sets = 30 798 choice sets across all respondents) and shows that the status quo was chosen 81% of the time, job A was chosen in 6.5% of the choice sets, and job B in 10.8%.

**Table 3:** Choice frequencies

Choice	Frequency	Percent
Not answered	501	1.6
Job A	2008	6.5
Job B	3339	10.84
Stay at my current job	24 950	81.01
Total choice sets	30 798	

The results of the GMNL model show this is preferred to a mixed logit model on the basis of the Bayesian Information Criteria (BIC) (results available on request). Table 4 shows a strong preference for the status quo (their current job) shown by the large negative coefficients for the constant terms. The statistically significant standard deviations for all attributes suggest that the strength of preference for their current job varies across specialists. The signs of the attributes are in the expected direction. Specialists prefer higher earnings, fewer hours, less on-call, more teaching and research opportunities, less administration, and working in metropolitan areas. For the continuous attributes of earnings, hours worked, percent time in the private sector and percent time in administration, we tested the linearity of each variable (one-by-one) by comparing against models where each of these attributes were re-coded in categories (i.e. non-linear). Likelihood ratio tests confirmed that for each of these attributes the hypothesis of linearity was not rejected.

**Table 4:** Results from GMNL model and marginal willingness to pay

	Mean (se)	SD	Marginal WTP^**d**^ (% annual income)	Marginal WTP (AUD$ annual income)
Change in earnings	0.096*** (0.004)	-		
Change in hours worked	− 0.106*** (0.005)	0.057*** (0.006)	− 1.10	− $3591
On call^a^: 1 in 4, frequently called out	− 0.196*** (0.049)	0.661*** (0.062)	22.24	$72 461
On call^a^: 1 in 4, infrequently called out	1.118*** (0.069)	0.747*** (0.071)	35.87	$116 885
On call^a^: 1 in 10, frequently called out	1.416*** (0.062)	0.797*** (0.099)	38.97	$126 976
Percentage of time in private practice	− 0.006*** (0.001)	0.021*** (0.001)	− 0.06	− $187
Teaching/research opportunities^b^: some	0.429*** (0.046)	0.811*** (0.060)	8.90	$28 988
Time spent in administration	− 0.065*** (0.007)	0.098*** (0.008)	− 0.67	− $2191
Location^c^: Metro-based	0.413*** (0.040)	0.425*** (0.037)	12.83	$41 808
Location^c^: Metro-based + option to visit	0.409*** (0.048)	1.042*** (0.070)	12.79	$41 662
Constant (job A)	− 4.262*** (0.097)	2.155*** (0.099)		
Constant (job B)	− 4.190*** (0.089)	1.886*** (0.110)		
Tau	1.038*** (0.050)			
Gamma	− 0.585*** (0.107)			
Log-Likelihood	− 12 833			
Number of observations	90 891			
Chi-sq. (df)	3030 (12)***			
AIC	25 716			
BIC	25 951			

*p < 0.1

**p < 0.05

***p < 0.01

^a^Reference category is ‘1 in 2 frequently called out’

^b^Reference category is ‘none’

^c^Reference category is ‘large regional centre’

^d^For categorical attributes marginal willingness to pay is not simply the ratio of coefficients because they are effects coded (see Scott et al. [28] for method)

**Fig. 2:**
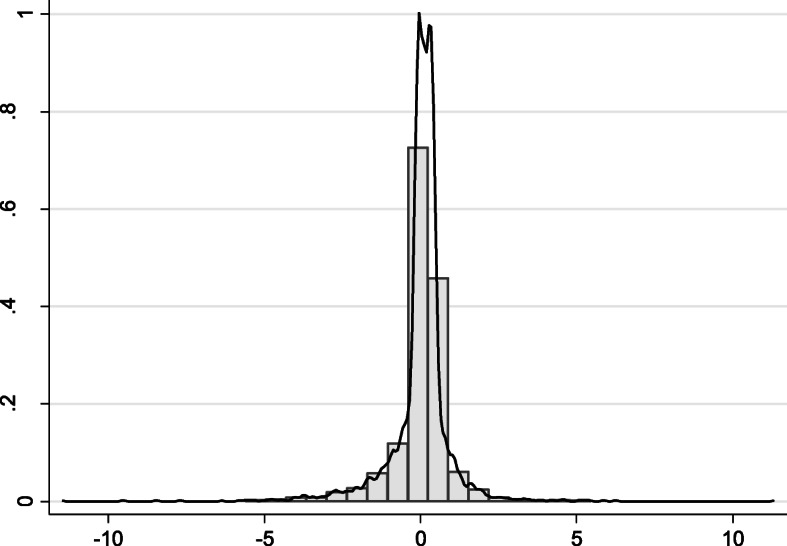
Distribution of standardised marginal utility of the percentage of time in private sector work. Notes: The *β*_*n*_ are distributed according to the distribution function *F*(*β*_*n*_; *θ*, *τ*) and are the expected values of *β*_*n*_ given the parameter estimates and the choices made by each individual: $E\left[{\beta}_n|{Y}_{n,}{X}_n;\hat{\theta},\hat{\tau}\Big)\right]$ [29]. Two doctors, who completed the same set of nine choices (*X*_*n*_) and choose the same alternatives (*Y*_*n*_), will have the same individual-specific estimate of the marginal utility *β*_*n*_ (24)

On average, specialists prefer to work in the public sector, shown by a negative marginal utility of the percentage of time spent in private practice. The coefficient measures the effect on utility of a 1% increase in the percentage of time spent in the private sector. The standard deviation suggests that the marginal utility varies across respondents. Since the coefficient presented is an average of individual specific marginal utilities, these individual marginal utilities were recovered and standardised to have a mean of zero and standard deviation of 1 and are plotted in Fig. 2. This has quite a tight distribution with the majority of respondents lying within ± 2 standard deviations of the mean. Fifty-nine percent of specialists prefer working in the private sector, whilst 41% prefer working in the public sector. However, those preferring the public sector do so more strongly than those preferring the private sector, such that the mean marginal utility of working in the private sector is negative.

The marginal rate of substitution between private sector work and earnings shows that for a 1% increase in the proportion of time spent in the private sector, specialists would need to be compensated 0.057% of their annual income, which is about $186. This can also be expressed in terms of working an extra session (4 h or half a day) in the private sector, representing a more realistic margin. With an average specialist spending 44% (19.95 h) of their 45.3 weekly working hours in the private sector, an increase of 4 h per week to 23.95 h represents an increase in the proportion of hours from 44 to 53%. This 9 percentage point increase would require them to be paid $1680 per year to maintain their utility. This is quite small compared to the average annual income of $325 000 and compared to the value of other attributes shown in the last two columns of Table 4.

### Is the preference for public sector work associated with specialists’ characteristics?

To investigate the factors influencing the variation in preferences for public sector work, the standardised measure of the marginal utility in Fig. 2 is used in an ordinary least squares (OLS) regression (Table 5). The independent variables focus on aspects of the life/career cycle and include age in 5-year bands, whether they have dependent children, and whether the respondent is an Australian graduate. Separate models are estimated for males and females. Overall, the explanatory power of these models is very low. For men, those aged 65–70 and approaching retirement have a stronger marginal utility for private sector work. This is also the case for women (aged 61–65 years old), but the effect is much stronger than for men. This is likely to reflect a preference for either boosting retirement income, or more likely a preference for less challenging work as doctors reduce their hours of work before they retire. In addition, female specialists with dependent children have a stronger preference for working more hours in the private sector. Since we have controlled for income, this is likely to reflect a preference for more autonomy and flexibility over working hours.

**Table 5:** Association of life/career cycle factors with the marginal utility of private sector work

	Males		Females	
	coef	se	coef	se
Aged 36–40	− 0.049	0.142	− 0.024	0.129
Aged 41–45	− 0.032	0.138	− 0.092	0.129
Aged 46–50	0.058	0.136	− 0.007	0.130
Aged 51–55	− 0.012	0.136	0.130	0.134
Aged 56–60	0.078	0.140	0.015	0.155
Aged 61–65	0.122	0.142	0.466**	0.197
Aged 65–70	0.257*	0.156	0.395	0.294
Aged 71–75	− 0.128	0.186	0.606	0.465
Aged 75–89	− 0.073	0.230	0.557	0.644
Has dependent children	0.064	0.057	0.163**	0.070
Australian medical school graduate	− 0.084	0.055	0.028	0.083
_cons	0.021	0.138	− 0.219	0.138
*N*	2237		870	
Adjusted *R*^2^	0.002		0.008	

**p < 0.05, *p < 0.1

We test whether income influences the preference for private sector work by splitting the sample into high- and low-income respondents and re-running the models (details and results in Additional file 1). These results suggest that the marginal utility of both earnings and private sector work are similar between those with low and high wages and that those with lower wages prefer the public sector and those with higher wages prefer the private sector though these associations are small.

### Is the preference for public sector work associated with risk attitudes?

Table 6 show the coefficients for the private practice attribute and its interaction with each risk aversion measure (full model results available on request). Note that the models including interaction terms have smaller sample sizes (see Additional file 1) since not all respondents to Wave 1 responded in Wave 2 (Big 5 risk aversion) or in Wave 6 (for risk aversion). The interaction term between the two overall measures of risk aversion and the private practice attribute are not statistically significant. The domain specific measures show that specialists who are more likely to take career risks prefer to spend more time in the public sector. Although career trajectories are more well-defined in the public sector, there is more tournament-type competition between specialists to work in major teaching hospitals to undertake high-quality research and teaching. This can create more uncertainty and competition when pursuing career options in the public sector. Risk averse doctors prefer the private sector.

**Table 6:** Preferences for time spent in private practice and risk aversion

	GMNL—base model (*n* = 41 889)		Big5 risk aversion (*n* = 41 889)		Overall risk aversion (*n* = 41 889)	
	Mean	sd	Mean	sd	Mean	sd
% time in private practice	− 0.0092***	− 0.0157***	− 0.0080***	0.0159***	− 0.0067***	0.0159***
x Big 5 risk aversion			0.0060	0.0016		
x Overall risk aversion					− 0.0018	− 0.0067***
	Career risks (*n* = 41 889)		Clinical risks (*n* = 41 889)		Financial risks (*n* = 41 889)	
	Mean	sd	Mean	sd	Mean	sd
% time in private practice	− 0.0040***	0.0159***	− 0.0056***	0.0189***	− 0.0087***	− 0.0013
x Career risk	− 0.0046***	0.0186***				
x Clinical risk			− 0.0053**	0.0047***		
x Financial risk					0.0004	0.0118***

Each GMNL model is the same as in Table 4, except for the addition of the single interaction term and reduced sample size

*p < 0.1

**p < 0.05

***p < 0.01

Taking clinical risks is also associated with a preference for more time in the public sector. Since the public sector treats more complex and challenging cases, it makes sense that it would attract doctors who prefer the greater challenges and uncertainty of treating such cases, which are likely to be patients most in need. The interaction term between the private practice attribute and taking financial risks is not statistically significant. Though there are some associations with risk aversion, the marginal utility of the private sector attribute remains statistically significant. However, the magnitude of the coefficient is around half that in the base model. This suggests that there is some evidence that risk attitudes in specific domains partly explain the observed preference for public sector work, though overall measures of risk attitudes were not statistically significant.

## Discussion

This paper provides new evidence on factors influencing the preferences of medical specialists for public or private sector work. After controlling for the key differences between public and private sector medical jobs, including earnings, as well as risk aversion, the results show only a weak preference towards spending more time in the public sector overall and among low wage earners, and a slight preference for time in the private sector among high wage earners. Doctors averse to clinical and career risk have a stronger preference for the private sector, contrary to the existing literature on public-private job choices, but reflecting the particular characteristics of physician’s jobs. Other job characteristics that differ between sectors are much more important to specialists than the amount of time spent in the public or private sector. This confirms our previous research using revealed preference data on hours worked that found little difference in the marginal utility of public and private sector work (Cheng et al. [16]).

These results suggest that non-wage factors play a stronger role in sector choice compared to wages and the sector itself. In Australia, medicine is the occupation delivering the highest earnings, and so the marginal utility of income for this group is likely to be small relative to lower earning occupations, and there may also be less variation in the marginal utility of income compared to other occupations. Our results also show that risk aversion is not only about financial uncertainty, but also about clinical and career uncertainty, and these may be more important drivers of behaviour.

The conclusions rest on the assumption that we have controlled for all other differences between public and private sector jobs. We included the most important job attributes from the literature, respondents were asked to assume that other factors were the same between jobs, and we examined the role played by risk attitudes. However, we cannot rule out other unobserved factors, though these are only likely to play a minor role. If important, they are likely to reduce the preference for public sector work to close to zero, strengthening our conclusions that it is the characteristics of the sector rather than a preference for working in the public sector.

A weak preference for the public sector may reflect the culture of medical practice in Australia. For some specialties where only public sector work is possible, or where the norm is dual practice, the amount of time spent in the private sector may be heavily influenced by specialty-specific norms. Our results are therefore likely to vary across specialties though sample sizes by specialty were too small, and there are no specific hypotheses about how the results might vary.

We did not include specific tests of ‘rationality’ or tests of continuity of preferences, such as identifying attribute non-attendance [30–32]. Though we cannot rule this out, unlike many other DCEs completed by patients or the general public where the choice tasks and attributes may be unfamiliar, our sample of doctors are highly educated and very familiar with the attributes presented and so could be less likely to provide ‘irrational’ responses, make errors, or ignore attributes and employ decision heuristics because of the difficulty of the choice task. Our use of a GMNL model does help to more flexibly model unobserved preference and scale heterogeneity compared to a simple mixed logit with uncorrelated coefficients, and so may capture differences in the error variances across individuals that can reflect the randomness of responses due to the adoption of different decision rules that are common to all coefficients [25].

Our response rate was 22.3% which is good for a sample of physicians [33]. There were some small differences in age, gender, hours worked, and geographic location, and we cannot observe representativeness with respect to other characteristics (unless they are correlated with observed characteristics) that may lead to bias. In particular, if more income-oriented physicians are less likely to fill out surveys and these are more likely to work in the private sector, then this could bias our results and underestimate the strength of preference for private sector work.

## Conclusions

In terms of policy conclusions, sector choice is more likely to be influenced by non-wage attributes of public and private sector jobs, and reductions in clinical uncertainty in public hospitals (e.g. through the use of clinical guidelines) could play a role. Specialists seem to be happy with their current balance of hours between the public and private sector. That may be largely because there is no regulation or restrictions about which sector they work in, and so their choices are optimal. Though choices might be optimal for specialists, they might not be for patients. Further research is required as to whether these choices are also socially optimal in terms of the effect of changes in doctors’ allocation of time between sectors on patients’ health outcomes and costs.

## Supplementary information

**Additional file 1.** The measures of risk aversion.

## Data Availability

De-identified MABEL data are available on application from the Australian Data Archive https://dataverse.ada.edu.au/dataverse.xhtml?alias=ada&q=MABEL. The dataset relating to this study that includes the discrete choice experiment is not included in the above due to confidentiality, but the data may be made available on request to the authors.

## Abbreviations

MABEL: Medicine in Australia: Balancing Employment and Life
GP: General practitioner
DCE: Discrete choice experiment
G-MNL: Generalised multinomial logit
AMPCo: Australasian Medical Publishing Company
BIC: Bayesian Information Criteria
OLS: Ordinary least squares
ASGC: Australian Standard Geographic Classification
WTP: Willingness to pay
