# Supplementary material for: Preferences of physicians for public and private sector work
Source: Hum Resour Health. 2020 Aug 10;18:59. doi: 10.1186/s12960-020-00498-4 (PMC7419199; doi:10.1186/s12960-020-00498-4)
Supplement: Supplementary file 1 — Additional file 1. The measures of risk aversion. [file 12960_2020_498_MOESM1_ESM.docx]

**Appendix 1.**

**The role of risk aversion**

We also examine risk attitudes which have been suggested as a reason why workers prefer the public sector to the private sector or to become self-employed (1-5) but have not been examined in the context of physician dual practice. Risk averse workers may be more likely to choose the public sector and are less likely to be self-employed because public sector jobs may have more job security and certainty of income. In a dual practice context, the role of risk attitudes may be more nuanced where the uncertainties of both sectors can be traded-off, compared to other jobs where only one sector can be chosen at a time. In our context, we hypothesise that specialists who are risk averse could prefer to work in, and spend more time in, the private sector. First, issues of job security and uncertainty of earnings are likely to be less important given high-income levels for physicians compared to other occupations, a fee-for-service payment system in the private sector with no price controls, and high demand for healthcare leading to little involuntary unemployment. Second, uncertainty in medical jobs is more likely to be related to uncertainty around diagnosis and how treatments provided to patients influence health outcomes (6). Clinical risk in the private sector may be lower because physicians can select which patients to treat and patients are less complex, more affluent and more homogeneous (7, 8). There is more certainty in the private sector of the type of work and skill that will be required, especially where there is more repetition of the same procedures, and where high volumes of single procedures is more likely to be rewarded, e.g. orthopaedic procedures, diagnostic tests, and cataract operations. The private sector may also offer more certainty of workload where it can more easily controlled. In comparison, public hospitals typically see more clinically and socially complex patients with more uncertainty about treatment outcomes and where physicians have no control of what patients they will see. The resource-constrained public sector may lead to unpredictable workload, peaks in demand and emergencies, more on-call and fewer resources to treat patients on time, such as availability of theatre space and staff shortages. So though risk aversion may matter, for non-GP specialists we hypothesise that risk averse specialists are more likely to prefer working in the private sector.

A domain-specific measure of risk attitudes was designed rather than a lottery as these have been shown to be highly correlated with one another (2) and are more easily included in a survey, and risk attitudes have also been shown to vary across different situations (Weber et al 2002). We adapted the Risk Propensity Scale (9) and included three domains specific to physicians working lives which were chosen because they related to some of the key areas examined in the MABEL survey: financial risk (e.g. investment with an uncertain outcome), career and professional risk (e.g. publicly challenging your professional colleagues), and clinical risk (e.g. recommending a treatment that is new, not usual practice or controversial). Respondents were asked “How likely are you to engage in each of the following activities (with score 1 being “very unlikely” and score 5 being “very likely”)?” An overall risk attitude measure was also used by summing the three individual scales based on the results of an exploratory factor analysis. Risk attitudes were measured for the first time in Wave 6 (2013). Data from Wave 6 was merged back to the DCE data in Wave 1. Here we assume that risk attitudes over time are highly correlated. This is a reasonable assumption since Schurer (10), using a large population sample in Australia, shows that risk aversion increases until around 40 years of age. After this age and especially for those who have middle to high incomes or in professional occupations it falls slightly but is generally stable until retirement. This does not rule out that risk aversion can be influenced by life events, but generally risk aversion is persistent and moderately stable (11). Note that most of our sample are over 40 years old, not yet retired, and in a high-income professional group, supporting our assumption of stability.

As a sensitivity check a second measure of risk aversion was derived from a measure of personality in Wave 2 (2009), using the Short 15-item Big Five Inventory (12) which comprises the five factors of extraversion, agreeableness, conscientiousness, neuroticism and openness to experience, each measured using three items scored on a seven point likert scale from ‘does not apply to me at all’ to ‘applies to me perfectly’. The personality traits of high openness to experience and extraversion, combined with low conscientiousness, neuroticism and agreeableness have been found to be closely associated with measures of risk attitudes (13). Risk-seeking individuals, who score above the median in the distribution on openness and extraversion and below the median on conscientiousness, agreeableness and neuroticism, are coded as one and others as zero. We merge the personality measures from Wave 2 into the Wave 1 data. In addition to evidence that risk aversion is stable in high income adult populations, the same is the case for personality traits (11, 14) and so we assume that this trait does not change after one year.

**Do preferences vary by income?**

We assume that one of the main motivators for working in the private sector is high income. Therefore it is likely that specialists with high hourly wages have a stronger preference for work in the private sector, which is where they generate their higher income. We run our model separately on two samples to test this: those earning more than $150/hour and those earning between $20 and $150 per hour. The correlation between private sector time is only modestly correlated with hourly wage (correlation = 0.4165, *p* < 0.001) and less within the sub-samples (low earners: correlation = 0.0860, *p* < 0.001; high earners: correlation = 0.2556, *p* < 0.001), suggesting other job characteristics may be more important. However, it is possible that, by virtue of choosing the status quo much more frequently than the other jobs, the results will show a stronger preference for the private sector among those spending more time in it. To address this concern, we run all analyses using the forced choice data using only scenarios A and B and excluding the status quo, and find no substantial differences in the results.

In the analyses, we are interested in differences in the *private* coefficients, and those on *earnings*, since these lend support to the profit-maximizing hypothesis. The results are shown in Table A1. There is a difference in the *private* coefficient: for high earners more private sector work increases utility (a 1% decrease in private sector time can be compensated with a 0.08% increase in income) and for low earners it lowers utility (a 1% increase in private sector time can be compensated with a 0.09% increase in income), and the magnitude of each is slightly larger than when the data are combined (0.057% compensation). There is little difference in the *earnings* coefficient. The marginal utility of an increase in earnings is higher for low income earners. Analysis using the forced choice data led to slightly different results, though with the same interpretation: the private coefficient was insignificant when the data were combined, and the compensating differential was 0.21% for high wage earners, so this sub-sample displayed a stronger preference for the private sector in the forced choice questions. The result for low wage earners was roughly the same. The earnings coefficients were similar across the forced choice analyses, though they were higher in magnitude for the status quo data across all samples.

These results suggest that the marginal utility of both earnings and private sector work are similar between those with low and high wages, and that those with lower wages prefer the public sector and those with higher wages prefer the private sector though these associations are small. This confirms results from a more formal discrete choice labour supply model that estimated wage elasticities for public and private sector work using the dame data. This found that although wages mattered in the allocation of hours between the sectors, the magnitude of the effects were relatively small with wage elasticities in the range of 0.16 to 0.51 (15).

**Table A1: Results from GMNL model and marginal willingness to pay, by hourly wage group**

|  | **High earners (> $150/hour)** | | | **Low earners (≤ $150/hour)** | | | |
| --- | --- | --- | --- | --- | --- | --- | --- |
|  | **Mean**  **(SE)** | **SD**  **(SE)** | **Marginal WTP^d^**  **(% annual**  **income)** | **Mean**  **(SE)** | | **SD**  **(SE)** | **Marginal WTP^d^ (% annual income)** |
| Change in earnings | 0.113^***^  (0.007) |  |  | 0.132^***^  (0.009) | |  |  |
| Change in hours worked | -0.133^***^  (0.010) | 0.077^***^  (0.011) | -1.18 | -0.124^***^  (0.009) | | -0.080^***^  (0.010) | -0.94 |
| On call^a^: 1 in 4, frequently called out | -0.315^***^  (0.104) | 0.865^***^  (0.101) | 24.21 | -0.252^***^  (0.095) | | 0.451^***^  (0.117) | 21.38 |
| On call^a^: 1 in 4, infrequently called out | 1.531^***^  (0.136) | 0.999^***^  (0.091) | 40.49 | 1.610^***^  (0.129) | | 0.684^***^  (0.082) | 35.53 |
| On call^a^: 1 in 10, frequently called out | 1.845^***^  (0.154) | 1.421^***^  (0.163) | 43.26 | 1.707^***^  (0.120) | | 1.209^***^  (0.118) | 36.26 |
| Percentage of time in private practice | 0.009^***^  (0.002) | 0.027^***^  (0.003) | 0.08 | -0.011^***^  (0.002) | | 0.025^***^  (0.003) | -0.09 |
| Teaching/research opportunities^b^: some | 0.240^***^  (0.063) | 0.564^***^  (0.068) | 4.24 | 0.689^***^  (0.117) | | 1.178^***^  (0.140) | 10.47 |
| Time spent in administration | -0.051^***^  (0.014) | 0.148^***^  (0.015) | -0.45 | -0.054^***^  (0.010) | | 0.110^***^  (0.011) | -0.41 |
| Location^c^: Metro-based | 0.521^***^  (0.085) | 0.671^***^  (0.089) | 13.42 | 0.844^***^  ((0.083) | | -0.669^***^  (0.077) | 14.34 |
| Location^c^: Metro-based + option to visit | 0.481^***^  (0.088) | 1.380^***^  (0.127) | 13.07 | 0.198^***^  (0.076) | | 1.340^***^  (0.121) | 9.43 |
| Constant (Job A) | -4.145^***^  (0.180) | 1.922^***^  (0.200) |  | -4.354^***^  (0.152) | | 2.191^***^  (0.164) |  |
| Constant (Job B) | -4.423^***^  (0.182) | 1.868^***^  (0.159) |  | -4.350^***^  (0.150) | | 1.987^***^  (0.155) |  |
| Tau | 1.177^***^  (0.072) |  |  | 1.277^***^  (0.067) | |  |  |
| Gamma | -0.143^**^  (0.056) |  |  | -0.461^***^  (0.080) | |  |  |
| Log-Likelihood | -3,471.137 | | | -6,499.785 | | | |
| Number of observations | 25,176 | | | | 45,732 | | |
| Chi-sq (df) | 805.36 | | | | 1,237.12 | | |
| AIC | 6,992.3 | | | | 13,049.6 | | |
| BIC | 7,195.6 | | | | 13,267.8 | | |
| ^***^ p<0.01, ^**^ p<0.05, ^*^ p<0.1. Note. a) reference category is ‘1 in 2 frequently called out’. b) reference category is ‘none’. c) reference category is ‘large regional centre’. d) for categorical attributes marginal willingness to pay is not simply the ratio of coefficients because they are effects coded. | | | | | | | |

**References**

1. Buurman M, Delfgaauw J, Dur R, Van den Bossche S. Public sector employees: Risk averse and altruistic? Journal of Economic Behavior & Organization. 2012;83(3):279-91.

2. Dohmen T, Falk A, Huffman D, Sunde U, Schupp J, Wagner GG. Individual Risk Attitudes: Measurement, Determinants, and Behavioral Consequences. Journal of the European Economic Association. 2011;9(3):522-50.

3. Falk A, Becker A, Dohmen T, Enke B, Huffman D, Sunde U. Global Evidence on Economic Preferences*. The Quarterly Journal of Economics. 2018;133(4):1645-92.

4. Caliendo M, Fossen F, Kritikos AS. Personality characteristics and the decisions to become and stay self-employed. Small Business Economics. 2014;42(4):787-814.

5. Zhao H, Seibert SE. The big five personality dimensions and entrepreneurial status: a meta-analytical review. Journal of Applied Psychology. 2006;91(2):259.

6. Eddy D. Variations in Physician Practice: The Role of Uncertainty. Health Affairs. 1984;3(2):74-89.

7. Tynkkynen L-K, Vrangbæk K. Comparing public and private providers: a scoping review of hospital services in Europe. BMC Health Services Research. 2018;18(1):141.

8. Cheng TC, Haisken-DeNew JP, Yong J. Cream skimming and hospital transfers in a mixed public-private system. Social Science & Medicine. 2015;132:156-64.

9. Nicholson N, Soane E, Fenton‐O'Creevy M, Willman P. Personality and domain‐specific risk taking. J Risk Res. 2006;8(2):157-76.

10. Schurer S. Lifecycle Patterns in the Socioeconomic Gradient of Risk Preferences. IZA Discussion Paper No. 8821. Bonn: IZA; 2015.

11. Schildberg-Hörisch H. Are Risk Preferences Stable? Journal of Economic Perspectives. 2018;32(2):135-54.

12. John OP SS. The Big Five trait taxonomy: History, measurement, and theoretical perspectives. In: Previn LA, John OP, editors. Handbook of personality: Theory and research. 2. New York: Guilford Press; 1999. p. 102-38.

13. Nicholson N, Soane E, Fenton‐O'Creevy M, Willman P. Personality and domain‐specific risk taking. Journal of Risk Research. 2005;8(2):157-76.

14. Cobb-Clark DA, Schurer S. The stability of big-five personality traits. Economics Letters. 2012;115(1):11-5.

15. Cheng TC, Kalb G, Scott A. Public, private or both? Analyzing factors influencing the labour supply of medical specialists. Canadian Journal of Economics/Revue canadienne d'économique. 2018;51(2):660-92.
